# Proteomic profiling of the human amniotic stem cell-highly abundant secreted proteins

**DOI:** 10.7717/peerj.19449

**Published:** 2025-06-03

**Authors:** Dan He, Yuqiang Cheng, Hongkai Lv, Anqi Geng, Jie Zheng, Lin Dang, Pengfei Li

**Affiliations:** 1 Laboratory of Animal Center, Medical Experiment Center, Shaanxi University of Chinese Medicine, Xianyang, Shaanxi, China; 2 Research & Development Institute, Northwestern Polytechnical University, Shenzhen, Guangdong, China; 3 Northwestern Polytechnical University, Sanhang Institute for Brain Science and Technology, Xian, Shaanxi, China; 4 Department of Encephalopathy, Affiliated Hospital of Shaanxi University of Chinese Medicine, Xianyang, Shaanxi, China

**Keywords:** Proteomics, Human amniotic stem cell, HAMSCs, HAECs, Secreted proteins, ANXA2

## Abstract

Amnion-derived stem cells exhibit several significant advantages, including low immunogenicity, anti-inflammatory, anti-fibrotic, and angiogenic properties, which have garnered considerable attention as a potential source for cell therapy. Numerous studies have demonstrated that proteins secreted by amniotic stem cells play a crucial role in facilitating regenerative processes and reflect the therapeutic benefits across various diseases. Secreted protein products from stem cells offer solutions to challenges in cell therapy, such as improving efficacy during *in situ* or intravenous administration. These products also hold significant clinical and commercial potential. Nonetheless, the establishment of stringent quality control standards for secreted proteins from both amniotic mesenchymal stem cells (AMSCs) and amniotic epithelial cells (AECs) continues to pose a significant challenge. In this study, the expression profiles of secreted proteins from AMSCs and AECs were comprehensively analyzed utilizing mass spectrometry-based proteomics. The 71 highly abundant proteins and their potential biological functions were further investigated. Moreover, we identified and confirmed 17 hub proteins. Notably, ANXA2 was observed to enhance the expression of pro-inflammatory cytokines in macrophages. These findings may inform quality control measures for secreted protein products derived from amniotic stem cells and provide valuable insights for future research on the functional aspects of the secreted proteome in amniotic stem cells.

## Introduction

Amniotic-derived stem cells (ADSCs) are one of the most promising cell types in regenerative medicine due to their abundant availability, low immunogenicity, and multipotent differentiation capabilities. Recent studies have demonstrated that ADSCs can secrete various paracrine factors and regulate the tissue microenvironment, thereby influencing target cell functions. These properties contribute to their significant therapeutic potential in the treatment and prevention of various diseases (Giampà et al., 2019; Lin et al., 2023; Papait et al., 2022a; Papait et al., 2022b; Ragni et al., 2021). Furthermore, studies indicate no significant difference in efficacy between fresh conditioned medium (CM) and freeze-dried products of amniotic stem cells (Silini et al., 2021). This approach facilitates the promotion and utilization of stem cell products, and crucially, may retain the same therapeutic capabilities as ADSCs. Previous studies have identified several secreted proteins with therapeutic potential and elucidated their roles in various diseases. AMSCs secrete insulin-like growth factor binding protein-3 (IGFBP-3), Dickkopf-related protein 3 (Dkk3), and Dickkopf-related protein 1 (DKK-1), which inhibit the activation of hepatic stellate cells (HSCs) by suppressing the Wnt/β-catenin signaling pathway, thereby alleviating liver fibrosis in mice (Liu et al., 2022). By secreting lysyl oxidase-like 2 (LOXL2), plasminogen activator inhibitor-1 (PAI-1), colony-stimulating factor (C-GSF), periostin, and tissue inhibitor of metalloproteinases-1 (TIMP-1), AMSCs facilitate the acceleration of skin wound healing (He et al., 2020; Li et al., 2019). Additionally, it has been shown that hAMSCs secrete various cytokines such as interleukin-6 (IL-6), osteopontin (OPN), angiopoietin-2 (ANG-2), hepatocyte growth factor (HGF), transforming growth factor- beta (TGF-β), angiopoietin-1 (ANG-1), interleukin-11 (IL-11), follistatin, galectin-1, macrophage migration inhibitory factor (MIF), insulin-like growth factor 2 (IGF-2), monocyte chemoattractant protein 1 (MCP-1), and interleukin-8 (IL-8). Similarly, hAECs can secrete brain-derived neurotrophic factor (BDNF), neurotrophin-3 (NT-3), vascular endothelial growth factor (VEGF), fibroblast growth factor (bFGF), and insulin-like growth factor (IGF), as well as transforming growth factor-beta 1 (TGF-β1). These factors play important roles in tissue development, neuronal regeneration, angiogenesis, and anti-apoptosis (He et al., 2020; Li et al., 2019; Wu et al., 2017).

These studies suggest that these proteins secreted by ADSCs possess considerable potential for application. However, the variability in the secreted proteins derived from amniotic tissues of different individuals, coupled with the relatively limited sources of collected samples, presents challenges for conducting effective quality control research on active components during the production process. In recent years, several studies have systematically analyzed the characteristics and potential functions of proteins secreted by AMSCs (Muntiu et al., 2023). Similarly, the role of CM derived from AECs in liver fibrosis has been investigated through proteomics analysis (Alhomrani et al., 2017). Our previous research also employed proteomics to examine the regulatory functions of CM from AMSCs and AECs in the context of wound healing (He et al., 2020). While these studies contribute valuable insights into the mechanisms underlying the therapeutic effects of the secretome from AMSCs and AECs, there remains a notable gap in understanding the regulatory functions of high-abundance proteins and the core roles of specific proteins. This limitation poses a challenge to advancing research in this domain. Therefore, systematically identifying high-abundance proteins with significant regulatory functions in the secretome of different sources of AMSCs and AECs remains a key issue in this field.

We conducted a systematic characterization of the secretomes of hAECs and hAMSCs derived from three distinct human sources. Utilizing bioinformatic and statistical analyses, we identified proteins that are commonly and highly abundant, and subsequently examined their associated biological functions. Among these secreted proteins, annexin A2 (ANXA2) emerged as a potentially pivotal regulator of innate immunity. Consequently, we employed qPCR to assess the impact of ANXA2 on the expression of inflammatory cytokines, including interleukin-6 (IL-6), interleukin-1β (IL-1β), tumor necrosis factor-alpha (TNF-α), and transforming growth factor-beta (TGF-β), in macrophages.

## Materials and Methods

### Sample source

Human monocytic cell line THP-1 was obtained from the Cell Bank of Chinese Academy of Sciences (Shanghai, China). THP-1 monocytes were cultured in RPMI 1640 medium (VivaCell, Shanghai, China), supplemented with 10% fetal bovine serum (VivaCell, Shanghai, China) and 1% penicillin–streptomycin (Solarbio) at 37 °C and 5% CO_2_. The THP-1 monocytes were differentiated to macrophages with 10 ng/ml phorbol-12-myristate-13-acetate (PMA) for 24 h. Proteins secreted by human amniotic epithelial cells (hAECs) and human amniotic mesenchymal stem cells (hAMSCs) were harvested and the peptide samples were analyzed by LC-MS/MS, as described previously (He et al., 2020).

### Global database search and protein quantification

All generated raw files were submitted to Maxquant (Cox & Mann, 2008) search engine (v.1.5.2.8) with label-free quantitation (LFQ) analysis. Tandem mass spectra were searched against human uniprot database concatenated with reverse decoy database. Trypsin/P was specified as cleavage enzyme allowing up to four missing cleavages. The precursor and fragment ion mass tolerance were set to five ppm and 20 ppm, and the mass tolerance for fragment ions was set as 0.02 Da. Carbamidomethyl on Cys was specified as fixed modification and acetylation modification and oxidation on Met were specified as variable modifications. FDR was adjusted to < 1% and minimum score for modified peptides was set > 40. The data were filtered to ensure high confidence peptide identification, requiring a minimum of at least two unique peptides per protein.

### Bioinformatic analysis

Gene Ontology (GO) enrichment, Kyoto Encyclopedia of Genes and Genomes (KEGG), and Reactome pathway analysis were performed using the Database for Annotation, Visualization, and Integrated Discovery (DAVID) (Sherman et al., 2022). A pathway with *P*-value < 0.05 was regarded as the significant pathway. Protein interactions were analyzed using STRING database (Snel et al., 2000) and the interactions with a combined score > 0.4 were selected to construct the PPI networks using the Cytoscape software. Following the construction of the PPI network using STRING (version 12.0), hub proteins were identified by sorting nodes based on closeness, degree, and betweenness centrality values using the CentiScaPe plugin (version 2.2) in Cytoscape (version 3.10.1). Hub proteins were defined as those with a closeness value greater than 0.0087, a degree value exceeding 24.26, and a betweenness centrality higher than 48.99. The Human Protein Atlas (HPA) (Uhlen et al., 2015) provided the immunohistochemistry staining of ANXA2 in human placenta and the core cell type table displays data for eight cell types that are found in many tissues.

### RNA and qPCRs

The THP-1 cells were treated as previously described. After 24 h of PMA treatment, 10 ng of annexin A2 (ANXA2; MCE, Monmouth Junction, NJ, USA) was added to each well and control group received an equivalent volume of solution (0.1% BSA). After the 24-hours treatment, the fresh cells were washed three times with PBS. Then THP-1 cells were lysed in one mL RNAiso Plus solution (Takara, Shiga, Japan) at room temperature for 10 min, followed by phase separation with chloroform. Follow the steps isolated according to the manufacturer’s instructions. RNA purity and concentration were assessed using a SpectraMax^®^ QuickDrop™ spectrophotometer (Molecular Devices, Sunnyvale, CA, USA). The purified RNA was then reverse transcribed into complementary DNA (cDNA) using the PrimeScript™ RT Kit (Takara, Shiga, Japan), the reaction system is 20 µL. The reverse transcription reaction included an initial incubation at 42 °C for 2 min to remove genomic DNA, followed by incubation at 37 °C for 15 min, and was concluded by inactivation of the reverse transcriptase enzyme at 85 °C for 5 s, store the products at −20 °C. Real-time quantitative PCR (qPCR) was conducted using a two-step method on a LightCycler^®^ 480 II system (Roche Diagnostics, Basel, Switzerland) with TB Green^®^ Premix Ex Taq™ II (Takara, Shiga, Japan). The qPCR cycling conditions were as follows: initial denaturation at 95 °C for 30 s, followed by 40 cycles of denaturation at 95 °C for 5 s and annealing/extension at 60 °C for 20 s, reaction system is 25 µL. The mouse GAPDH gene served as the endogenous control. All qPCR reactions were performed in quadruplicate, and data were analyzed using the 2^−ΔΔCq^ method. The primer sequences are provided below.

**Table:** 

**Gene**	**Primer**
IL-6	Forward: 5′-AGACAGCCACTCACCTCTTCAG-3′
	Reverse: 5′-TTCTGCCAGTGCCTCTTTGCTG-3′
IL-1β	Forward: 5′-CCACAGACCTTCCAGGAGAATG-3′
	Reverse: 5′-GTGCAGTTCAGTGATCGTACAGG-3′
TNF-α	Forward: 5′-CTCTTCTGCCTGCTGCACTTTG-3′
	Reverse: 5′-ATGGGCTACAGGCTTGTCACTC-3′
TGF-β	Forward: 5′-TACCTGAACCCGTGTTGCTCTC-3′
	Reverse: 5′-GTTGCTGAGGTATCGCCAGGAA-3′
GAPDH	Forward: 5′-GTCTCCTCTGACTTCAACAGCG -3′
	Reverse: 5′-ACCACCCTGTTGCTGTAGCCAA -3′

### Statistical analysis

Statistical analysis was performed using the Graphpad Prism 7 software. The data are represented as the means ± standard error (S.E.) of at least three independent experiments. The samples were compared using the Student’s t tests and a *P*-value < 0.05 was considered statistically significant.

## Results

### Secretomic characterization of the amniotic membrane-derived cells

**Figure 1:**
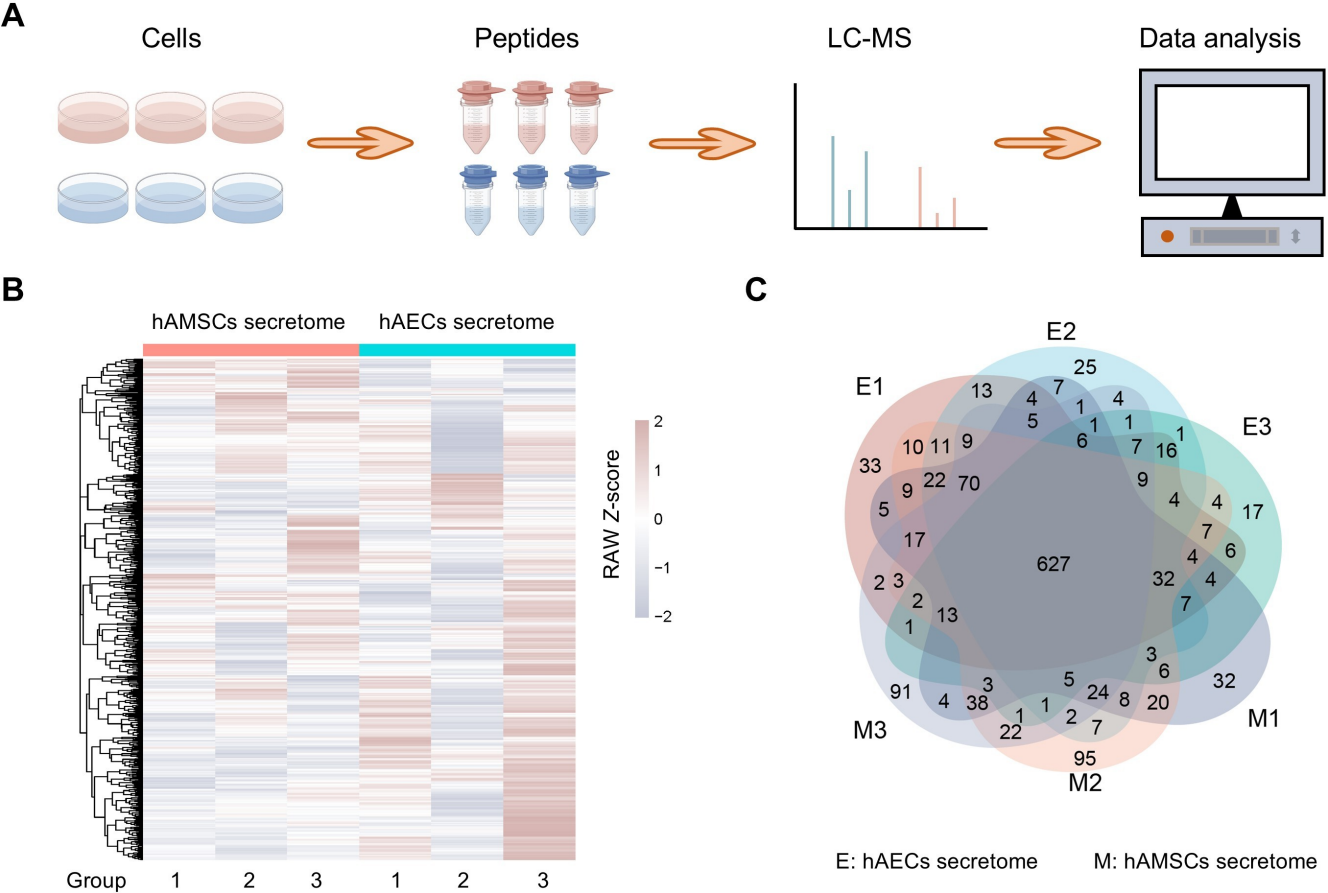
Proteome profiles and quantification of proteins among different amniotic membrane-derived cells secretome. (A) Workflow of the proteomic study, including the protein extraction, peptide enrichment, liquid chromatography-mass spectrometry (LC-MS) data generation, and data analysis. (B) Venn diagram resulting from grouping analysis of the secreted proteins identified in the hAECs and hAMSCs pools 1-3. (C) Heatmap of quantified proteins. Groups indicate three biological repeats under each condition. The sign (+/−) of the Z-score indicates the relative level of expression, and a higher Z-score corresponds to a greater expression level.

The hAMSCs and hAECs secretome were selected for analysis of label-free quantitative proteomics (Fig. 1A), more information is in Table S1. After filtering these proteins, a total of 1,401 proteins were considered eligible for further quantitative analysis and these quantified proteins in the six samples are shown in a heatmap (Fig. 1B). The rows of the heatmap are hierarchically clustered using Euclidean distance and complete linkage. Grouping analysis revealed a total of 627 proteins shared by all the samples and may be associated with the biological activities exerted by hAMSCs and hAECs secretomes (Fig. 1C). The overlapping region in the Venn diagram highlights the highly expressed proteins shared among all six sample groups, which helps to rule out the impact of inter-sample variation on the results.

### Evaluation of the most abundant proteins in the secretome

**Figure 2:**
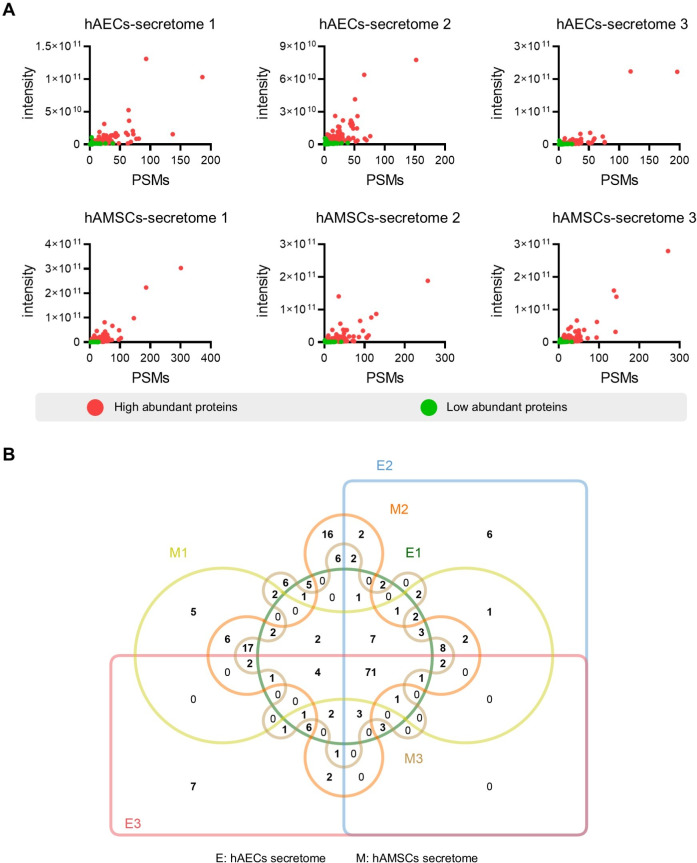
Identification of the high abundant proteins in the different secretome. (A) Scatter plot showing the distribution of the abundant proteins in the hAECs and hAMSCs pools 1-3. (B) Venn diagram of the high abundant proteins among the hAECs and hAMSCs pools 1-3.

To screen for the most abundant proteins, we applied a filter to the protein intensity and peptide-spectrum matches (PSMs) data. Highly abundant proteins were defined as intensity ≥ 1.5 ×10^9^, PSMs ≥ 5 and identified with at least two unique peptides (Fig. 2A). Specifically, we identified 142 (119), 163 (120), and 143 (108) the most abundant proteins in the different hAMSCs and hAECs secretomes. Finally, we identified the 71 commonly highly abundant proteins in the secretomes (Fig. 2B), which represent a conserved subset of proteins unaffected by inter-sample variation.

### Functional analysis of the highly abundant proteins

**Figure 3:**
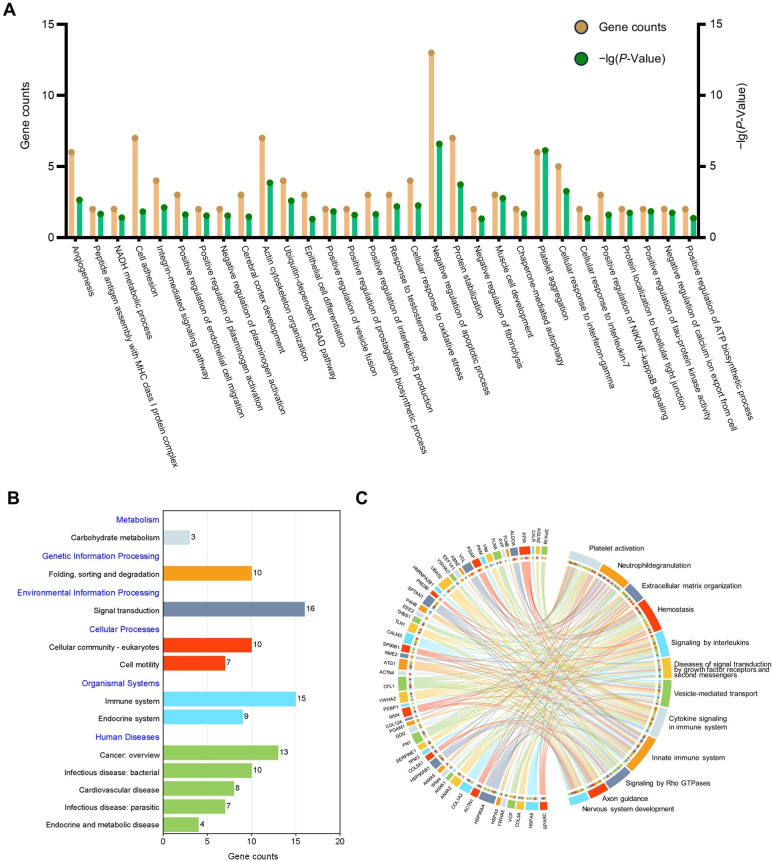
Functional enrichment analysis of the high abundant proteins. (A) GO enrichment analysis of the high abundant proteins. (B) KEGG pathways analysis of the high abundant proteins. The vertical axis represents the level two classification of KEGG pathway. Gene counts refer to the number of genes associated with each GO or KEGG pathways. (C) Reactome pathway analysis of the high abundant proteins. All pathways examined exhibit gene *p*-values below the threshold of 0.05.

GO analysis was undertaken to facilitate understanding of the biological significance of the 71 highly abundant proteins (Fig. 3A), the results showed that these proteins are mainly related to negative regulation of apoptotic process, cell adhesion, and inflammation-related pathways. To find out the important pathways of these proteins in the amniotic membrane-derived cells, we further analyzed the enrichment of the KEGG pathways through the KEGG database and conducted a secondary classification of all KEGG pathways (Fig. 3B). The results indicated that the highly abundant proteins were mainly involved in carbohydrate metabolism, folding, sorting and degradation, signal transduction, cellular community-eukaryotes, cell motility, immune system, endocrine system, cancer, infectious disease, cardiovascular disease, and endocrine and metabolic disease. Reactome pathway analysis showed that the highly abundant proteins were mainly associated with innate immune system (Fig. 3C).

### Selection of hub proteins in secretome

**Figure 4:**
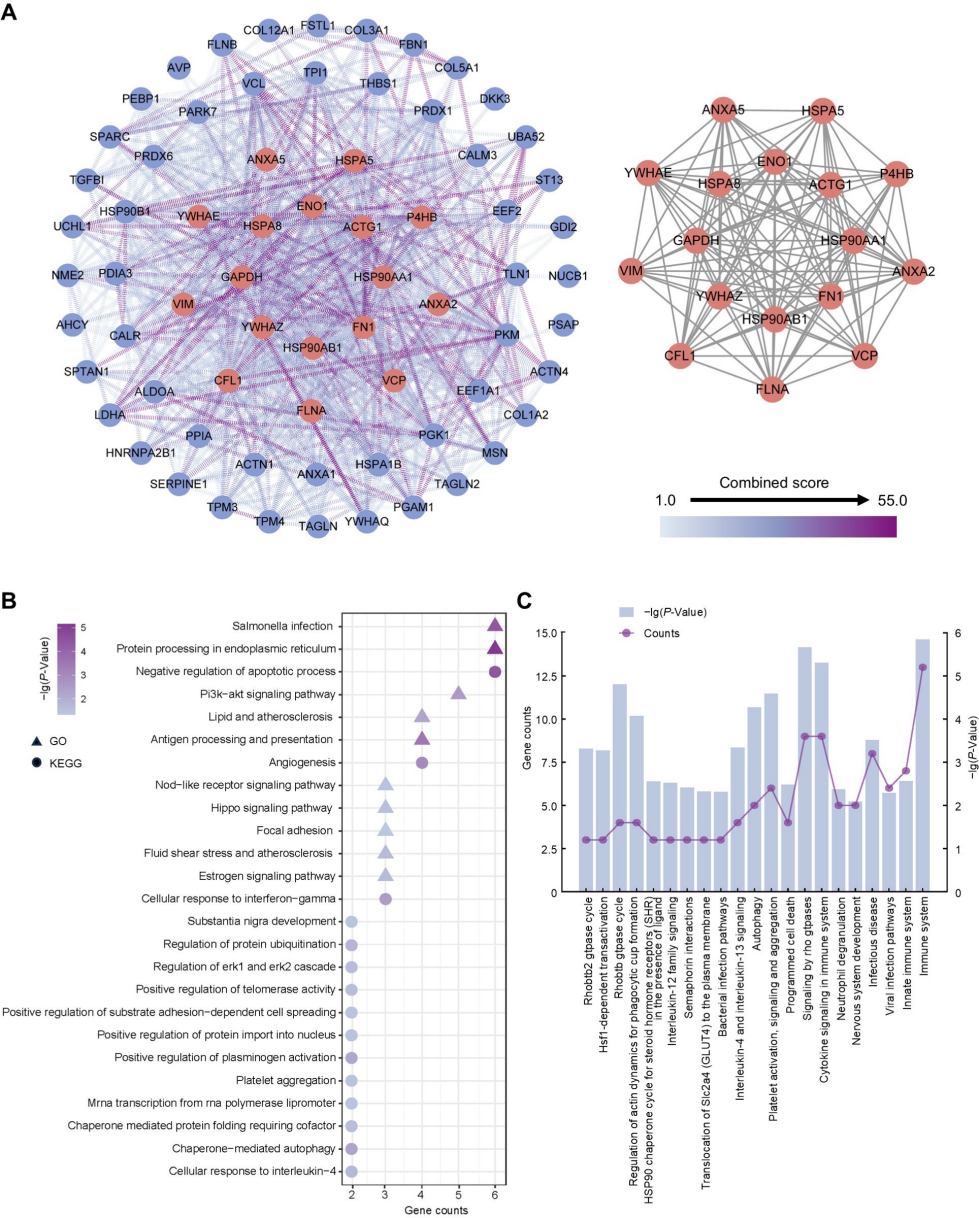
Establishment of protein-protein interaction network and functional enrichment analysis of the hub proteins. (A) Network of the high abundant proteins (left) and network of highly interconnected proteins (right). Hub proteins were defined as those with a closeness value greater than 0.0087, a degree value exceeding 24.26, and a betweenness centrality higher than 48.99. (B) GO and KEGG pathways analysis of the hub proteins. (C) Reactome pathway analysis of the hub proteins. Gene counts refer to the number of genes associated with each GO or KEGG pathways.

To ascertain hub proteins in secretome of the amniotic membrane-derived cells, we investigated the interaction and physiological connections of the 71 highly abundant proteins. Based on the PPI results, 69 of these had interactions, and the highly interacting proteins included ACTG1, ANXA2, ANXA5, CFL1, ENO1, FLNA, FN1, GAPDH, HSP90AA1, HSP90AB1, HSPA5, HSPA8, P4HB, VCP, VIM, YWHAE, YWHAZ (Fig. 4A). GO and KEGG analysis showed that the 17 proteins were concentrated in negative regulation of apoptotic process, angiogenesis, cellular response to interferon-gamma, protein processing in endoplasmic reticulum, salmonella infection, and PI3K-Akt signaling pathway (Fig. 4B). Reactome pathway analysis showed that these proteins were most associated with immune system (Fig. 4C).

### ANXA2 is the major protein secreted by the amniotic membrane-derived cells

**Figure 5:**
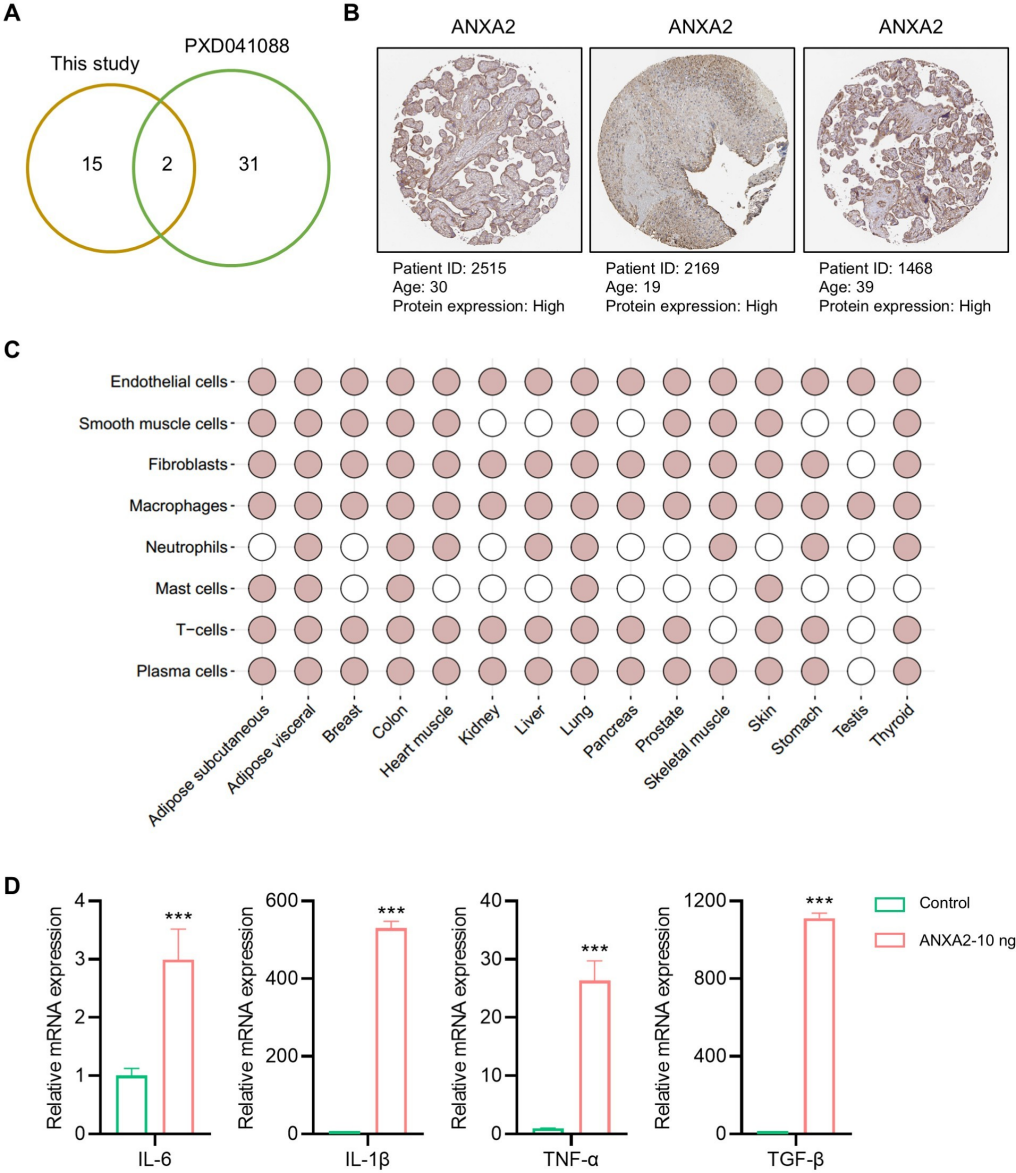
Tissue distribution and function of ANXA2. (A) Hub proteins identified currently comparing with the previous study. (B) Expressions of ANXA2 in human placenta. (C) Tissue specific and cell specificity of ANXA2. Colored dots indicate that the selected gene has core cell type specificity in the indicated tissue. Grey dots indicate that cell type is present within that tissue, but the selected gene is not predicted to be enriched there. (D) Validation of inflammatory factors mRNA expression in macrophages after ANXA2 treatment. All data are presented as mean ± SEM, *n* = 3. ^∗∗∗^*P* < 0.001, compared with control.

To further verify the reliability of the hub proteins, we downloaded and re-analyzed the proteomics data (PXD041088) hAMSCs secretome from public datasets, and compared them with the 17 proteins identified in our study. The comparison results showed that two (FLNA and ANXA2) of 17 proteins identified in this study were also identified in the previous study (Fig. 5A). Since only ANXA2 is reported as a secreted protein, it is the major protein secreted by the amniotic membrane-derived cells. In humans, amniotic membrane-derived cells originate from a placenta, and the over-expressions of ANXA2 in human placenta were also confirmed by using the immunohistochemistry (IHC) staining data from publicly available resources (Fig. 5B). In addition, public database also showed that ANXA2 was widely present in various cells of different organs, especially in endothelial cells and macrophages (Fig. 5C). The above findings suggested that ANXA2 may be involved in immune regulation by acting on macrophages. Therefore, we further verified the effect of exogenous ANXA2 on macrophages by qPCR, and the results showed that the inflammatory factors including IL-6, IL-1β, TNF-α, and TGF-β significantly increased after ANXA2 treatment (Fig. 5D).

## Discussion

The CM derived from amniotic stem cells (including AMSCs and AECs), and their freeze-dried products promote tissue repair and homeostasis by modulating immune responses, enhancing cell migration and differentiation, and inhibiting fibrosis (Silini et al., 2021). Furthermore, ADSCs secrete free soluble proteins and extracellular vehicles (EVs), which serve as crucial mediators in tissue repair and disease treatment (Papait et al., 2022a; Papait et al., 2022b). Secreted protein products offer significant potential to overcome challenges in ADSC clinical translation, including: (1) stringent laboratories and specialized technical personnel: The production of amniotic cells necessitates laboratories that comply with good manufacturing practice (GMP) standards. These cells must be cryopreserved in liquid nitrogen, and subsequently revived and cultured prior to therapeutic application. These cells must be cryopreserved in liquid nitrogen, subsequently revived, and cultured before clinical application. The duration and conditions of cryopreservation, as well as the seeding density post-revival, significantly impact MSC viability. Although karyotype analysis is performed on MSC batches to ensure quality, it remains insufficient to fully guarantee the stability of cellular functions (Giri & Galipeau, 2020; Jayaraman et al., 2021). Additionally, the laboratory’s proximity to the treatment site is crucial to prevent cell apoptosis during transportation; (2) Limited Cell Supply and Immunological Risks: For optimal vitality amniotic cells from passages 1 or 2 are typically utilized in clinical treatments. It is imperative that cells from the same tissue source are used simultaneously (Sanz-Nogués & O’Brien, 2021; Solomon et al., 2016). When the availability of amniotic tissue is inadequate or the quantity of cells extracted from the tissue is insufficient for a single treatment, the therapeutic intervention cannot be maintained. For optimal vitality, ADSCs from passages 1 or 2 are typically utilized in clinical treatments, and it is imperative to use cells from the same tissue source simultaneously (Sanz-Nogués & O’Brien, 2021; Solomon et al., 2016). When the availability of amniotic tissue is inadequate or the quantity of cells extracted is insufficient for a single treatment, the therapeutic intervention cannot be sustained. Although MSCs exhibit low immunogenicity, xenogeneic MSCs may still trigger specific immune responses (Griffin et al., 2013). (3) High clinical risk/limited effectiveness for some indications: Research has demonstrated that amniotic stem cells can inhibit tumor cell proliferation, induce apoptosis, and ameliorate chemotherapy-induced ovarian fibrosis and follicular closure (Jantalika et al., 2023). Despite evidence indicating that amniotic stem cells are not tumorigenic, controversy persists (Jafari et al., 2021). Additionally, in pathological conditions such as stroke, Huntington’s disease, and Alzheimer’s disease, ADSCs have demonstrated promising therapeutic effects. However, the ability of these cells to traverse the blood–brain barrier remains uncertain, resulting in a significant disparity between clinical and laboratory research outcomes (Giampà et al., 2019; Kim, Suh & Chang, 2020; Lin et al., 2023). Secretome-based products derived from ADSCs hold promise in addressing these challenges. These extracellular factors offer significant advantages in storage and transportation, effectively reducing logistical costs (Marolt Presen et al., 2019). Moreover, due to the relatively low concentration of cytokines in CM, their clinical use does not induce tissue inflammatory responses, unlike recombinant proteins (Katagiri et al., 2013), and their ease of use is comparable to drugs like insulin (Cañas-Arboleda et al., 2025). Notably, the therapeutic efficacy of CM from the first and fifth passages of ADSCs is comparable, and mixed cultures from different human sources show no significant differences (Silini et al., 2021). Furthermore, the expression levels of secreted proteins and related chemokines/inflammatory cytokines remain consistent across different cell densities, indicating highly uniform immunomodulatory properties among different batches of MSCs. This uniformity effectively avoids issues such as inconsistent cell activity caused by elevated senescence markers at low seeding densities (Cañas-Arboleda et al., 2025; Torre et al., 2015). During the actual production process, cells from the same batch can be passaged and expanded on a large scale, thereby reducing the variability introduced by cells derived from different tissue sources. The secretory factor product is devoid of viable cells, thereby eliminating the risk of cell colonization and tumorigenesis. It has been demonstrated that CM can inhibit tumor cell proliferation and induce apoptosis, both *in vitro* and *in vivo* (Jafari et al., 2023; Jantalika et al., 2023; Riedel et al., 2019).

MSCs are rich in neuroregulatory factors that promote the proliferation and differentiation of neural stem cells, offering a promising strategy for treating or delaying diseases associated with neuronal death or neural stem cell depletion, such as Alzheimer’s disease and Parkinson’s disease (Hasanpour et al., 2022; Mendes-Pinheiro et al., 2019; Mendes-Pinheiro et al., 2023; Santamaria et al., 2021; Teixeira et al., 2017; Zhuo et al., 2024). Additionally, the secretome of MSCs can mitigate neuroinflammation caused by brain injuries such as stroke, traumatic brain injury (TBI), and cerebral hemorrhage through immunomodulatory effects, thereby improving patient outcomes (Asgari Taei et al., 2022; Dzhauari et al., 2023; Jha et al., 2022; Silvana et al., 2024). Furthermore, proteins or EVs within the CM are more likely to reach the lesion site *via* the bloodstream. Notably, the immunomodulatory capacity of secretory factors in CM is significantly higher than that of EVs (Papait et al., 2022a; Papait et al., 2022b). These secretory proteins demonstrate significant advantages and market potential for clinical applications.

Despite the promising market potential and convenience of stem cell secretome-based products, common challenges associated with MSC-derived products must be addressed. First, the heterogeneity of MSC sources and cells themselves (Costa et al., 2021), along with the complexity of CM preparation and derivative processing (*e.g.*, lyophilized powder), underscores the importance of standardized production processes and regulations (Bari et al., 2018). To ensure consistency in the secretome profile, producing CM derivatives under strict GMP standards is critical (Bari et al., 2018; Gimona et al., 2017; Mendicino et al., 2014). This is a prerequisite for evaluating and comparing clinical efficacy and facilitates quality control (Phelps et al., 2018). However, this remains a formidable task. Beyond standardization, the choice of culture medium significantly impacts secretome-based products. Unlike fetal bovine serum (FBS) commonly used in laboratories, clinical production often employs human platelet lysate (hPL) to avoid cross-contamination and xenogeneic rejection risks. However, hPL is costly to produce and carries potential disease transmission risks. Developing well-defined, stable, cost-effective, and cell-growth-supportive media compliant with pharmaceutical production standards is essential for MSC clinical applications (Hoang et al., 2021; Sagaradze et al., 2019). Over the past decade, companies like Thermo Fisher Scientific and Yocon China have developed suitable media tailored to the tissue origin of stem cells (Sagaradze et al., 2019). To ensure accurate reference for clinical ADSCs, further research is needed. However, due to proprietary and confidentiality issues, the composition of different media brands is not disclosed, potentially leading to discrepancies in efficacy comparisons across laboratories and introducing risks in process optimization. Moreover, pathological microenvironments vary, and cytokines from lesion tissues may interact with MSCs, influencing product efficacy (Barnhoorn et al., 2022; Sagaradze et al., 2019). Therefore, merely profiling and analyzing the secretome of MSCs from different tissue sources is insufficient. Further exploration of the differences in indications and mechanisms of action among secretome-based products is required (Hodgson-Garms et al., 2025; Trigo et al., 2024). This may lead to an exciting future where patients receive “customized” products with optimized efficacy, laying the foundation for combination therapies. However, the absence of standardized quality control measures for assessing CM produced by different laboratories and methods remains a major barrier to clinical standardization and large-scale implementation. In addition to technical challenges, significant financial investment, the need for skilled personnel (scientists and clinical evaluators), regulatory approvals, and varying authorization requirements across countries pose inevitable hurdles to advancing this field (Sanz-Nogués & O’Brien, 2021).

Our proteomic analysis identified several highly abundant secreted proteins, including FLNA, TAGLN2, and Col3a1 (Fig. 3C). FLNA deficiency in cortical pyramidal neurons disrupts RAC1 expression and cofilin phosphorylation, reducing dendritic spine generation and leading to periventricular nodular heterotopia (Falace et al., 2024). Additionally, a complete knockout of FLNA leads to embryonic lethality, accompanied by severe defects in cardiac structure development (Feng et al., 2006). The knockout of the Col3a1 gene in mice results in exhibit excessive neuronal migration beyond the pial basement membrane during brain development, leading to causing cortical lamination disorders (Luo et al., 2011). TAGLN2 is highly expressed in bone marrow mesenchymal stem cells (BMSCs), and its knockout disrupts osteogenic differentiation disorder (Kuo et al., 2011).

GO analysis also revealed an enrichment of cellular responses to oxidative stress (Fig. 3A). Oxidative stress-induced damage to retinal pigment epithelial cells (RPECs) and retinal ganglion cells (RGCs) is a significant contributor to various eye diseases. Exosomes derived from AECs and AMSCs are enriched with cytokines that mitigate H_2_O_2_ and hypoxia-induced damage to RPECs, promote their proliferation, and consequently reverse retinal layer atrophy in glaucomatous rats (Seong et al., 2023). These factors have demonstrated significant therapeutic potential in disease treatment and prevention through actions such as immune response inhibition, promotion of angiogenesis, anteng et al. 2024). Additionally, AMSCs-CM accelerates wound healing in second-degree burned mice by activating the PI3K/AKT pathway, inhibiting apoptosis in skin cells, and promoting cellular proliferation (Li et al., 2019). Our study also identified a high abundance of secreted proteins from ADSCs, which exhibit anti-apoptotic and oxidative stress functions, corroborating our findings.

The potent immunomodulatory capabilities of AMSCs and AECs, along with their secreted proteins, have garnered widespread recognition. Pathway enrichment analysis further revealed a significant association between these highly abundant proteins and the regulation of signaling by interleukins, cytokine signaling in immune system, and innate immune system (Fig. 3C). CM derived from AM and AMSCs has been shown inhibits T cell proliferation decrease Th1 cell populations, reduces monocyte differentiation into mature dendritic cells, and suppress their polarization towards the pro-inflammatory M1 macrophage phenotype while promoting an anti-inflammatory M2 phenotype (Silini et al., 2021). The application of CM can facilitate wound healing and diminish the expression of pro-inflammatory cytokines and markers such as IL-6, IL-8, TNFα, ICAM1, and TLR2 (Takahashi et al., 2021). These finding suggest that ADSCs can modulate immune cell activity to suppress the expression of pro-inflammatory factors. Additionally, a screening of core proteins was conducted for 71 highly abundant proteins, followed by a joint analysis utilizing data from the public database on the secretome of AMSCs (PXD041088) to ascertain the universality of key proteins. Through this analysis, we identified ANXA2 as a universally highly abundant protein.

Notably, ANXA2 does not function as an immunosuppressive factor in the conventional sense. Instead, ANXA2 is a significant member of the annexin family, characterized as a Ca2+-regulated phospholipid and membrane-binding protein (Gerke, Creutz & Moss, 2005). ANXA2 plays a significant role in innate immune cells such as macrophages, monocytes, and dendritic cells, participating in the regulation of various stages of inflammation and exerting either anti-inflammatory or pro-inflammatory effects. In the initial phase of the immune response, ANXA2 plays a critical role in maintaining vascular integrity by limiting vascular permeability, thereby preventing edema and the extravasation of blood cells. This function inhibits the recruitment of leukocytes and the release of inflammatory mediators (Heyraud et al., 2008; Luo et al., 2017). Subsequently, ANXA2 facilitates the repair of the intracellular membrane system, modulates the activation of inflammasomes, and promotes the formation of autophagosomes, which are essential for the clearance of damaged organelles and pathogens (Moreau et al., 2015). Studies involving ANXA2 knockout mice have demonstrated an increased susceptibility to the dissemination of Pseudomonas aeruginosa infection to the lungs and other organs (Li et al., 2015). ANXA2 plays a role in the infection and replication processes of cells or viruses. It mediates the recognition and response to pathogens such as bacteria or viruses by influencing the activation of the NLR family CARD domain-containing protein 4 (NLRC4) inflammasome through pattern recognition receptors (PRRs) (Zhang et al., 2022; Taylor, Skeate & Kast, 2018; Wang et al., 2016). ANXA2 interacts with viral proteins and is upregulated, thereby suppressing IFN-β and interferon-stimulated response element (ISRE) activity, which in turn promotes viral proliferation (Zhang et al., 2025). In chronic inflammatory environments, ANXA2 facilitates sustained angiogenesis, which contributes to diseases like diabetic retinopathy (Dallacasagrande et al., 2023; Huang et al., 2011). Therefore, ANXA2 may serve as a critical target for influencing viral infection and antiviral proliferation, warranting further research and development of its inhibitors. Beyond its role in immune regulation, ANXA2 plays significant roles in the fibrinolytic system, tissue repair, and fibrosis. Injection of ANXA2 recombinant protein at the tibial fracture site in mice promotes endochondral ossification and accelerates healing (Wang et al., 2025). Interestingly, ANXA2 activation or upregulation is associated with fibrosis (Zheng et al., 2025). The absence of ANXA2 affects tissue-type plasminogen activator (tPA)-dependent plasmin generation on endothelial cell surfaces, which may contribute to cerebral venous thrombosis and systemic lupus erythematosus, leading to thrombus formation (Ao et al., 2011; Cesarman-Maus et al., 2011; Fassel et al., 2021). ANXA2 can be targeted in various pathological processes, including viral infection, tissue healing acceleration, and antithrombotic therapy. However, due to the diverse functions of ANXA2 under different pathological conditions and its varying expression locations (extracellular matrix, cell membrane, and cytoplasm), as well as its critical roles in multiple tissues, interventions targeting ANXA2 may cause side effects in patients with different indications. Additionally, ANXA2 exhibits distinct functions under different immune states, influencing both monocyte differentiation and recruitment (Lim & Hajjar, 2021), making it challenging to ensure treatment stability across individuals. To effectively harness the advantages of ANXA2, in-depth research and analysis of its specific mechanisms in different diseases are necessary to enable precise interventions or target its downstream key proteins for specific modulation.

Treatment of macrophages with ANXA2 significantly increased the expression of inflammatory factors, including IL-6, IL-1β, TNFα, and TGF-β. This suggests that ANXA2 may play a pro-inflammatory role. AMSCs and AECs, along with their secreted proteins, are well documented for their immunosuppressive properties. This observation indicates that the regulatory functions of amniotic secreted proteins extend beyond immunosuppression. Indeed, during pregnancy, intra-amniotic infection is a major cause of preterm birth and premature rupture of fetal membranes, and infection of the amniotic fluid can also adversely impact the development of the fetal nervous system and lungs (Muntiu et al., 2023). The amnion may exhibit pro-inflammatory functions to counteract infection. AECs express significant levels of β-defensins, which are notably upregulated in response to lipopolysaccharide (LPS) stimulation (Buhimschi et al., 2004; Szukiewicz et al., 2016). Furthermore, both AECs and AMSCs express a variety of functional Toll-like receptors, which play an important regulatory role in innate immunity (Gillaux et al., 2011; Sato et al., 2016). Additionally, it has been documented that AECs can secrete pro-inflammatory cytokines such as IL-6 and IL-8 to maintain the homeostasis of the amniotic fluid environment (Yu, 2015). These studies indicate that ADSCs may contribute to the stabilization of the body’s lesion environment by secreting pro-inflammatory factors, thereby clearing potential pathogens, and t subsequently restoring environmental stability through the inhibition of immune cells. However, the regulatory effects of immune-activating factors derived from ADSCs in disease contexts have not yet been systematically studied, and the specific functions of these proteins in amniotic CM still require further validatio.

We identified ANXA2 as a representative secretory factor with dual immunomodulatory functions. Preliminary findings revealed that ANXA2-treated macrophages exhibited elevated mRNA expression levels of inflammatory factors such as IL-6, IL-1β, TNF-α and TGF-β, suggesting an immune-activated state. However, the specific role of extracellular ANXA2 in regulating immune homeostasis and disease progression requires systematic analysis in vivo. We hypothesize that ANXA2 secreted by ADSCs may interact with macrophages to promote tissue repair, while macrophages initiate inflammatory responses and release cytokines to help clear pathogens, phagocytize cellular debris, and promote the proliferation of target cells to accelerate healing. Our previous research has demonstrated that CM from ADSCs can effectively enhance wound healing (He et al., 2020). Therefore, we plan to knock down ANXA2 expression in ADSCs to produce ANXA2-deficient CM and treat skin wound model mice with recombinant ANXA2 protein at different concentration gradients. We will observe wound healing progression and histopathological changes at various time points. Flow cytometry will be used to monitor changes in macrophage surface markers during the healing process, along with inflammatory indicators. Additionally, we will investigate the immunomodulatory and anti-fibrotic effects of ANXA2 *in vivo*, as well as its impact on macrophage polarization during wound healing. In summary, our research aims to identify proteins that are highly abundant in ADSCs, with the aim of providing research ideas for the selection of preclinical translational quality control products for amniotic secreted proteins. Additionally, our findings indicate that the secreted protein ANXA2 is highly expressed in our samples, and corroborated by data from other databases. Preliminary results suggest that ANXA2 may enhance the expression of inflammatory factors in macrophages, implying that ADSCs could exhibit both immunoactivity and immunosuppressive functions. However, it is important to acknowledge that the primary limitation of this study is the limited number of amniotic samples analyzed. To obtain more precise and comprehensive information, large-scale analyses of ADSCs involving a substantial number of tissue sources are required. This approach ensures the selection of proteins that are both feasible and universally valuable. However, our investigation did not encompass other core proteins, and the functional study of ANXA2 was limited to *in vitro* experiments utilizing recombinant proteins. Consequently, this does not directly demonstrate that ADSCs can activate immune responses through the secreted protein ANXA2, but rather indicates only a partial effect of ANXA2 on macrophages. Therefore, further research is warranted and will be pursued in future studies.

## Conclusions

This study conducted a comprehensive analysis of the high-abundance secreted proteins from AMSCs and AECs, utilizing mass spectrometry-based proteomics and subsequently identifying hub proteins. The results suggest that the immune-activation factor ANXA2 may be pivotal within the secretome of amniotic stem cells. These findings contribute valuable insights for the quality control of proteomic products derived from amniotic stem cells and propose a potential avenue for further research into the immune regulatory functions of these secreted proteins.

##  Supplemental Information

10.7717/peerj.19449/supp-1 Supplemental Information 1 Secreted proteins identified and quantified from the global proteome of human amniotic stem

10.7717/peerj.19449/supp-2 Supplemental Information 2 Information on qPCR

10.7717/peerj.19449/supp-3 Supplemental Information 3 Raw Data of qPCR

10.7717/peerj.19449/supp-4 Supplemental Information 4 MIQE checklist

## Additional Information and Declarations

### Data Availability

The following information was supplied regarding data availability: The mass spectrometry data are available at Proteomexchange via the PRIDE partner repository: PXD057076.
