# Supplementary material for: Proteomic profiling of the human amniotic stem cell-highly abundant secreted proteins
Source: PeerJ. 2025 Jun 3;13:e19449. doi: 10.7717/peerj.19449 (PMC12143284; doi:10.7717/peerj.19449)

**Supplementary data on qPCR**

1. **Nucleic acid extraction**

From RNA extraction to qPCR, use RNase- and DNase-free pipette tips, Eppendorf tubes, PCR tubes, and 8-well strips throughout. Before starting the experiment, wipe the work surface with an RNase inhibitor.

The A260/A280 ratio of extracted between 1.9 and 2.1.

RNA quantification is shown in the table below:

| **Sample name** | **Concentration** | **A260/230** | **A260/280** |
| --- | --- | --- | --- |
| Control | 155.2ug/µL | 0.856 | 2.019 |
| ANXA2-10ng | 150.4ug/µL | 1.715 | 2.021 |

1. **Justification of reference genes**

In this study, we have chosen to use GAPDH as the housekeeping gene.

Firstly, GAPDH is a housekeeping gene that is widely distributed in cells across various tissues and is abundant in cells, accounting for 10%-20% of the total protein content. The GAPDH gene has highly conserved sequences and is expressed at high levels in almost all tissues. The expression of GAPDH mRNA and protein in the same type of cells is generally constant, making GAPDH one of the most commonly used housekeeping genes^[1]^.

Secondly, studies have indicated that the expression level of GAPDH itself is not affected by LPS treatment. This suggests that during the differentiation of macrophages, the function and activity of GAPDH may change, but the expression level remains stable. Therefore, GAPDH can serve as the internal control gene for this study^[2,3]^.

References:

1. Barber RD, Harmer DW, Coleman RA, Clark BJ. GAPDH as a housekeeping gene: analysis of GAPDH mRNA expression in a panel of 72 human tissues. Physiol Genomics. 2005 May 11;21(3):389-95.
2. Galván-Peña S, Carroll RG, Newman C, Hinchy EC, Palsson-McDermott E, Robinson EK, Covarrubias S, Nadin A, James AM, Haneklaus M, Carpenter S, Kelly VP, Murphy MP, Modis LK, O'Neill LA. Malonylation of GAPDH is an inflammatory signal in macrophages. Nat Commun. 2019 Jan 18;10(1):338.
3. Maess MB, Sendelbach S, Lorkowski S. Selection of reliable reference genes during THP-1 monocyte differentiation into macrophages. BMC Mol Biol. 2010 Dec 1;11:90.
4. **Data analysis**

LightCycler® 96 Software’s, ver.1.2.

In qPCR, replicate wells with a standard deviation greater than 0.4 are considered outliers and are excluded from the statistical analysis.

1. **qPCR Oligonucleotides**

**IL6 Human qPCR Primer Pair**

Sequence accession number:

BC015511, NM_000600, NM_000600.1, NM_000600.2, NM_000600.3, NM_000600.4, BT019748, BT019749, NM_000600.5

The primer seqence is from Origene, CAT#：HP200567;

The sequece:

Forward：5'-AGACAGCCACTCACCTCTTCAG-3'

Reverse: 5'-TTCTGCCAGTGCCTCTTTGCTG-3'

PCR product size：132bp

Location of amplicon: chr7:22727554+22728743

Amplicon length: 1190bp

Amplicon seqence:

AGACAGCCACTCACCTCTTCAGTTCTGCCAGTGCCTCTTTGCTGAGACAGCCACTCACCTCTTCAGaacgaattgacaaacaaattcggtacatcctcgacggcatctcagccctgagaaaggaggtgggtaggcttggcgatggggttgaagggcccggtgcgcatgcgttccccttgcccctgcgtgtggccgggggctgcctgcattaggaggtctttgctgggttctagagcactgtagatttgaggccaacggggccgactagactgacttctgtatttatcctttgctggtgtcaggaagttcctttcctttctggaaaatgcagaatgggtctgaaatccatgcccacctttggcatgagctgagggttattgcttctcagggcttccttttccctttccaaaaaattaggtctgtgaagctcctttttgtcccccgggctttggaaggactagaaaagtgccacctgaaaggcatgttcagcttctcagagcagttgcagtactttttggttatgtaaactcaatggctaggattcctcaaagccattccagctaagattcatacctcagagcccaccaaagtggcaaatcataaataggttaaagcatctccccactttcaatgcaaggtattttggtcctgtttggtagaaagaaaagaacacaggaggggagattgggagccc

acactcgaattctggttctgccaaaccagccttgtgatcttgggtaaattccctaccacctctggactccatcagtaaaattgggcgtggactaggtgatctcatagatccttcctgctggaacattctatggcttgaattatattctcc

taattattgtcaaaattgctgttattaagtatctactgtgtgccaggcactttaaataaatattgtgtctaatcttcaaaacaaatttgcaaggaaggtttttggagataaggaaactgagactcaggattaagtaacacacctaaagtcacaggtgagcttggaactgaacccaagtgtgcccccactccactggaatttgcttgccaggatgccaatgagttgtagcttcatttttcttagagactttcctggctgtggttgaacaatgaaaaggccctctagtggtgtttgttttagggacacttaggtgataacaattctggtattctttcccagacatgtaacaagagtaacatgtgtgaaagCAGCAAAGAGGCACTGGCAGAA

The melting peak of this primer.


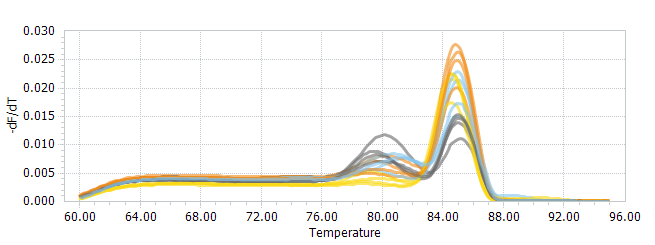


Using BLAST to silico specificity:


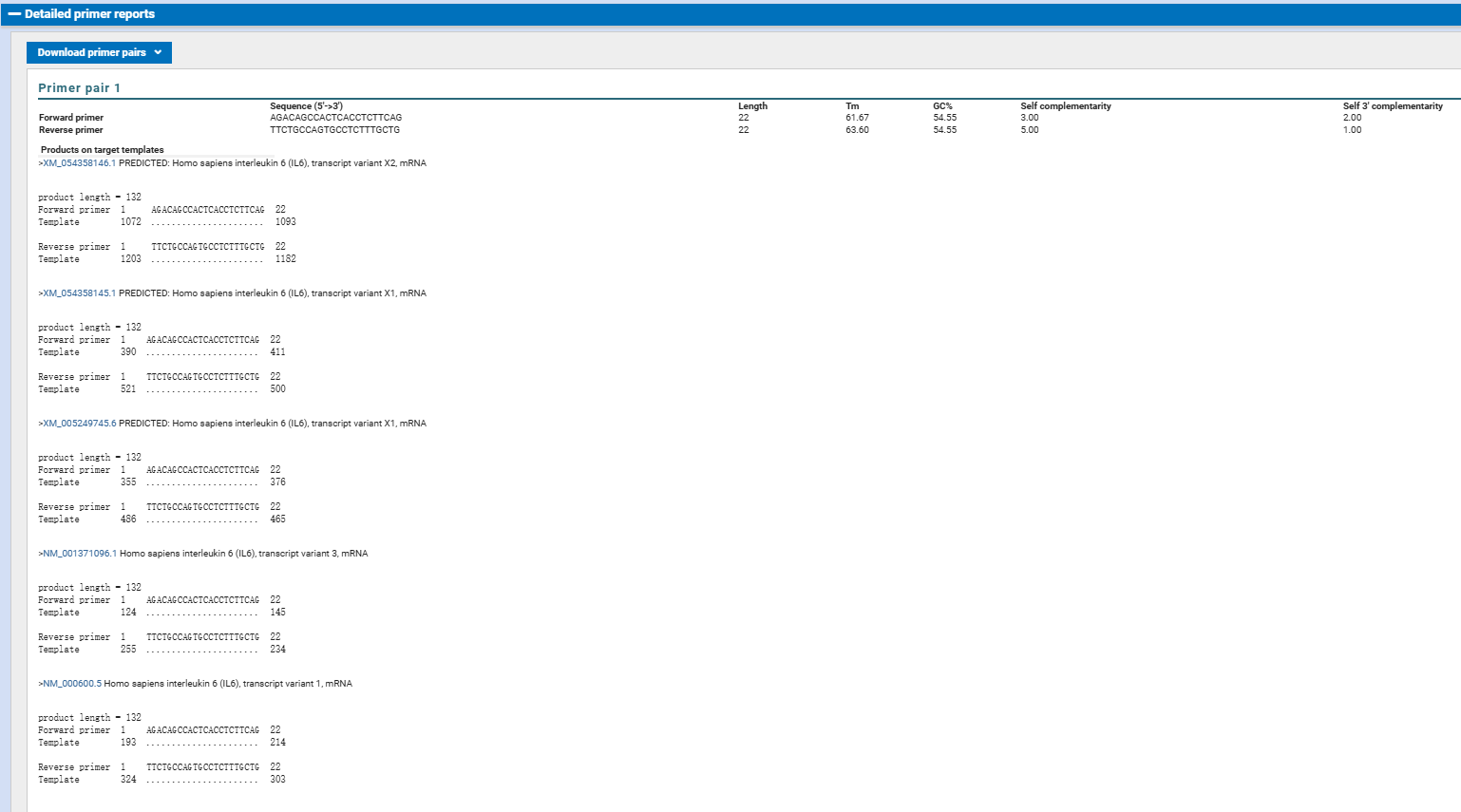


The Amplification curves:


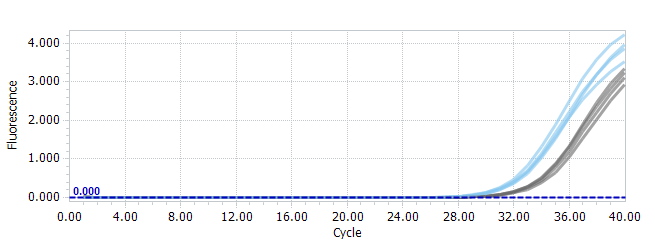


**IL-1β Human qPCR Primer Pair**

Sequence accession number:

NM_000576, NM_000576.1, NM_000576.2, BC008678, BC008678.1, BT007213, NM_000576.3

The primer seqence is from Origene, CAT#：HP200544;

The sequece:

Forward：5'- CCACAGACCTTCCAGGAGAATG-3'

Reverse: 5'- GTGCAGTTCAGTGATCGTACAGG-3'

PCR product size: 131bp

Location of amplicon: chr2:112832754-112833431

Amplicon length: 678bp

Amplicon seqence:

CCACAGACCTTCCAGGAGAATGGTGCAGTTCAGTGATCGTACAGGCCACAGACCTTCCAGGAGAATGacctgagcaccttctttcccttcatctttgaagaaggtagttagccaagagcaggcagtagatctccacttgtgtcctcttggaagtcatcaagccccagccaactcaattcccccagagccaaagccctttaaaggtagaaggcccagcggggagacaaaacaaagaaggctggaaaccaaagcaatcatctctttagtggaaactattcttaaagaagatcttgatggctactgacatttgcaactccctcactctttctcaggggcctttcacttacattgtcaccagaggttcgtaacctccctgtgggctagtgttatgaccatcaccattttacctaagtagctctgttgctcggccacagtgagcagtaatagacctgaagctggaacccatgtctaatagtgtcaggtccagtgttcttagccaccccactcccagcttcatccctactggtgttgtcatcagactttgaccgtatatgctcaggtgtcctccaagaaatcaaattttgccgcctcgcctcacgaggcctgcccttctgattttatacctaaacaacatgtgctccacatttcagaacctatcttcttcgacacatgggataacgaggcttatgtgcacgatgcaCCTGTACGATCACTGAACTGCAC

The melting peak of this primer.


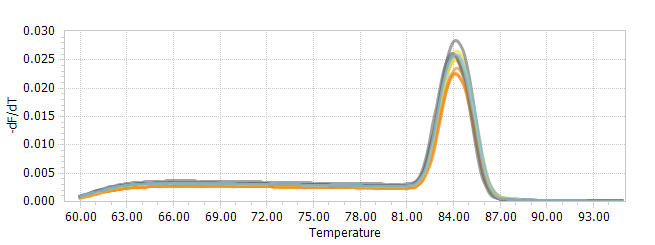


Using BLAST to silico specificity:


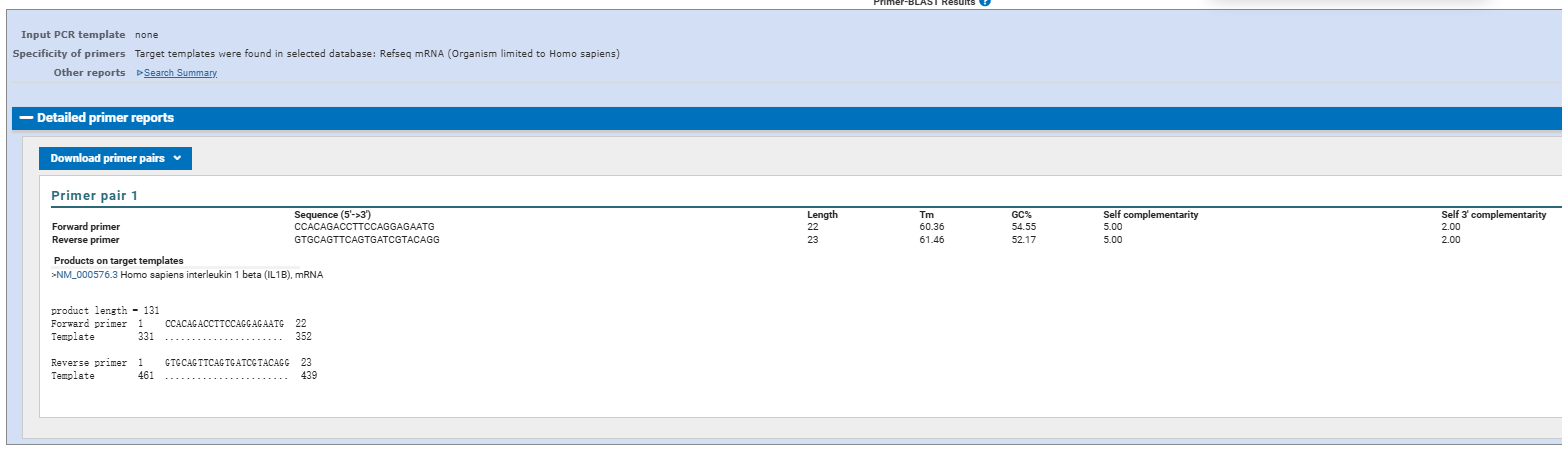


The Amplification curves:


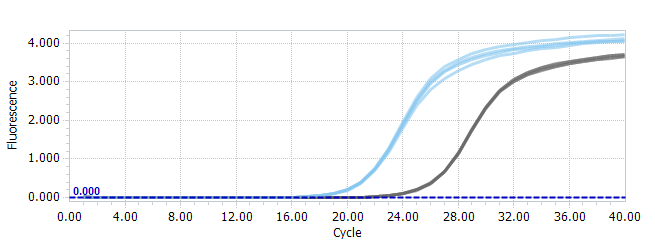


**TNF-α Human qPCR Primer Pair**

Sequence accession number:

NM_000594, NM_000594.1, NM_000594.2, NM_000594.3, BC028148, BC028148.1, BI908079, BP215875, NM_000594.4

The primer seqence is from Origene, CAT#：HP200561

The sequece:

Forward：5'-CTCTTCTGCCTGCTGCACTTTG-3'

Reverse: 5'-ATGGGCTACAGGCTTGTCACTC-3'

PCR product size：135bp

Location of amplicon: chr6:31575880+31576807

Amplicon length: 928bp

Amplicon seqence:

CTCTTCTGCCTGCTGCACTTTGATGGGCTACAGGCTTGTCACTCCTCTTCTGCCTGCTGCACTTTGgagtgatcggcccccagagggaagaggtgagtgcctggccagccttcatccactctcccacccaaggggaaatggagacgcaagagagggagagagatgggatgggtgaaagatgtgcgctgatagggagggatggagagaaaaaaacgtggagaaagacggggatgcagaaagagatgtggcaagagatggggaagagagagagagaaagatggagagacaggatgtctggcacatggaaggtgctcactaagtgtgtatggagtgaatgaatgaatgaatgaatgaacaagcagatatataaataagatatggagacagatgtggggtgtgagaagagagatgggggaagaaacaagtgatatgaataaagatggtgagacagaaagagcgggaaatatgacagctaaggagagagatgggggagataaggagagaagaagatagggtgtctggcacacagaagacactcagggaaagagctgttgaatgcctggaaggtgaatacacagatgaatggagagagaaaaccagacacctcagggctaagagcgcaggccagacaggcagccagctgttcctcctttaagggtgactccctcgatgttaaccattctccttctccccaacagttccccagggacctctctctaatcagccctctggcccaggcagtcagtaagtgtctccaaacctctttcctaattctgggtttgggtttgggggtagggttagtaccggtatggaagcagtgggggaaatttaaagttttggtcttgggggaggatggatggaggtgaaagtaggggggtattttctaggaagtttaagggtctcagctttttcttttctctctcctcttcaggatcatcttctcgaaccccGAGTGACAAGCCTGTAGCCCAT

The melting peak of this primer.


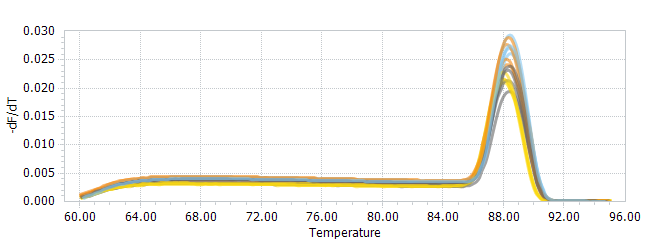


Using BLAST to silico specificity:


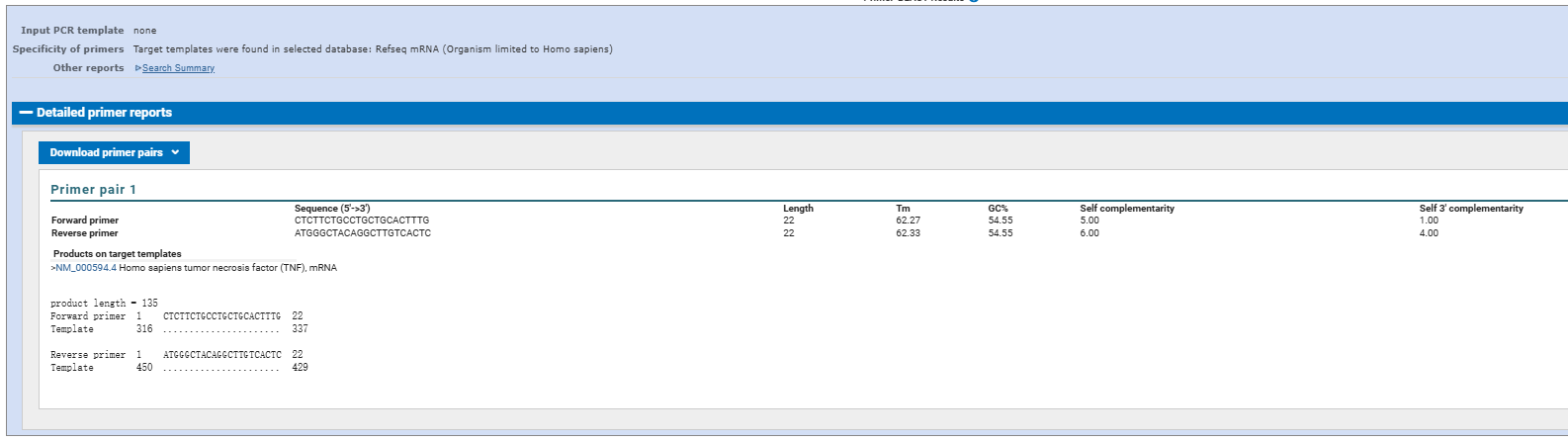


The Amplification curves:


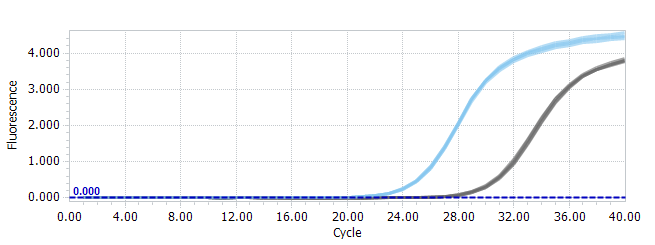


**TGF-β Human qPCR Primer Pair**

Sequence accession number:

NM_000660, NM_000660.1, NM_000660.2, NM_000660.3, NM_000660.4, NM_000660.5, NM_000660.6, BC000125, BC000125.1, BC001180, BC022242, BG115704, BM692012, BP267938, BT007245, NM_000660.7

The primer seqence is from Origene, CAT#：HP200609

The sequece:

Forward：5'- TACCTGAACCCGTGTTGCTCTC-3'

Reverse: 5'- GTTGCTGAGGTATCGCCAGGAA-3'

PCR product size：122bp

Location of amplicon: >chr19:41344829-41348380

Amplicon length: 3552bp

Amplicon seqence:

TACCTGAACCCGTGTTGCTCTCGTTGCTGAGGTATCGCCAGGAATACCTGAACCCGTGTTGCTCTCccgggcagagctgcgtctgctgaggctcaagttaaaagtggagcagcacgtggagctgtaccaggtgaggacatgagccagaaggaaggtcagggcatgggctggagagggtgagctgtgaccaagggggtggctgtgggtcggctggttacaaggtccacctagatggtccctgaaggatagaagaacacaaaccatacaatcctagaacgctttttttttttttttgagacagtgtctcactctgttgccaagctggagtgcagtggtatgacctcagctcactgcaatctccgcctcctgggttccagtgattctcctgcctcagcctcccaagtagctgggattacaggtgtgcgccaccacgcccagctaatttttgtatttttggtagagatggggttttaccatgttggccaggatggtctcgatctcctgacctcgtgatccacctgcctcggtctcccaaagtgctcagattacaggcgtgagccactgcgcctggcctcttttttttttttttttttttttgagatggagtcttgctctgtcgcccaggctggagtacaatggggtgatctcggctcactgcaacctccgcctcctgggttcaagtgattctcctgcctcagccacccgagtagctgggattacaggcatgtgccaacacgctgggctaatttttgtatttttagtagagacagagttttatcatgttggccaggcttgtctcaaactcctgccctcaagtgattctcctacctgagcctcccagagtgctgggattacaggtgtgagccactgcacccggaacctagagcacttttaaatgttcagactctttgcatcctaggatgttaaacacttagaaggctggaatcttaggagttggactctttaaggacacaggattcttgaaagttggaatctctgaaaaggttgggggctctagaatcattctgtccaatatgacagccactagtcacatttagcttgattaaaatttaaattgtttaaaattaaattaaaaatttagggccaggcatagtggctcacacctctaatcccagaactttgggaagccgaggcaggcagattgcttgagcccaggagtccgagaccagtctgggcaacatgttgaaacttcgtctctacaaaaaatactaaaattagcctgttatggtgatgcgtgcctctagtcccagctactcagtagtctgaggtgggaggattgattgagcccaggaggtcgaggctgtagtgaactgtgattgcaccactgcattccagcctgggtgatggagcgagaccgtctcaaaaatatatatatataggccaggcgtggtggctcatgcctgcaatcccagcactttgggaggccagggtgggtggatcacttgaggtcaggagttcaagaccagcctggccaacatgacgaaactccatctctactaaaaatacaaaaattagccaggtgtggtggcaggcacctgtaatcccagctacttgggaggctgaggtgggagaatcgcttgaccccaggaggtggagactgcagtgagcggagatcataccact

gcactccagcctgggcaacagagcgagactctgtctcaaaaaaataaaaatgaaaaagaaatcctcattctcactggccatatttctagtactctatagtcacatgtggttagcagctactattttggatgttgcagataaagcacatttccagaaagttctttaggacagcactgctctagaagatagggagcttccaagaggactggggcatctggaagggctggaggctctagcagtttctatgagctagaatccatatcagagggaatgttaactcataggatggtagatttcagactttcacagtgagagaactttgtcctatgttagcttggcttcttggagtctggggaattcagctttattctgcagtccctggagtggactatccagccccagaaaattcttcttttttttgtgtgtgtgtgtgatggagttttgctcctgttgcccaggctggagtgcaatggcaccatcttggctcaccacaacctttgcttcctgggttcaagcgattctcctgcctcagccacccgagcagctgggattacaggcatgcaccaccacacctagctaattttgtatttttagtagagacggtgtttctccatattggtcaggctggtctcgaacttctgacctctggtgatccaccctcctcggcctcccaaagtgctgggattacaagcgtgagcgactgtgcctggcccagaaaattattctacacagatgttgggtacctgcagcatctaggtgcggcatggcagactcttgggtttatagaacattagtcttctaaagcttctcaggctctagaagtggaaatcttgggatattatcaaacatttgaatcacaaaatgactttttttttttttgagacagagtctcactctgtcaccctggctagagtgcagtggtgcaatctcggctcactgcaacctctgtgtcttgggttcaagtgattctcctgcctcagcctcctgagtagctgggattacaggcgtgtgccaccacgcctggctaatttttgtatttttagtagagacgaggtttcactttgttggccaggctggtcttgaactcctgacctcaggagatccacccgccttggcctcccaaagtgctgggataacaggcgtgagccaccgtgcccagccggaatcattagaaatgacttctaagttactgagaattcagggtgtcaaatttgcagaaccgtatgctcagtaaaccccagaatgtttgcagcagaattttattttatttattattattattttgagacgaagtctcgtcctgtcgcccaggctggagtgcagtggcgtgatcttggctcactgcagtctgtgtctcccgggctcaagcaattctcctgcctcagcctcccaaatagtggcgactacaggcgtgtgccaccatgcctggctaatttttgtatttttagtagagatggggtttcactatgttggccaggctggtcttgaactcctgacctcaagtgatccacccaccttgggctcccaaagtgctgggattacaggcgtgagccactgcgcccggcccagaatgttagcagcagaattttagcattgtgggctgtccacgctgagtggggcttagcatttcaccaatgaggaaacaggcctcgagaaggcaagaaaacaccttcggctgagctgtgtgaaggtgacttgaccgcagcctgagctttttctccacccctcctctcatgggtactgttggggaggatgggtgccacaggaccacacaggtggctgtctgagagggtagtgcctgggaactttctggaagcctgtttggggaagcagatggggtgaaggattca

gttagtgtatgtggggtcgtgacaccatctacccactgtctctctcctgccttcatcatcctctagaaatacagcaacaaTTCCTGGCGATACCTCAGCAAC

The melting peak of this primer.


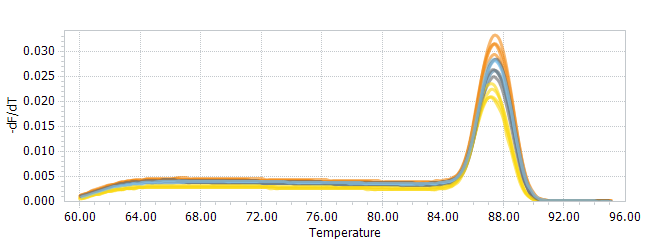


Using BLAST to silico specificity:


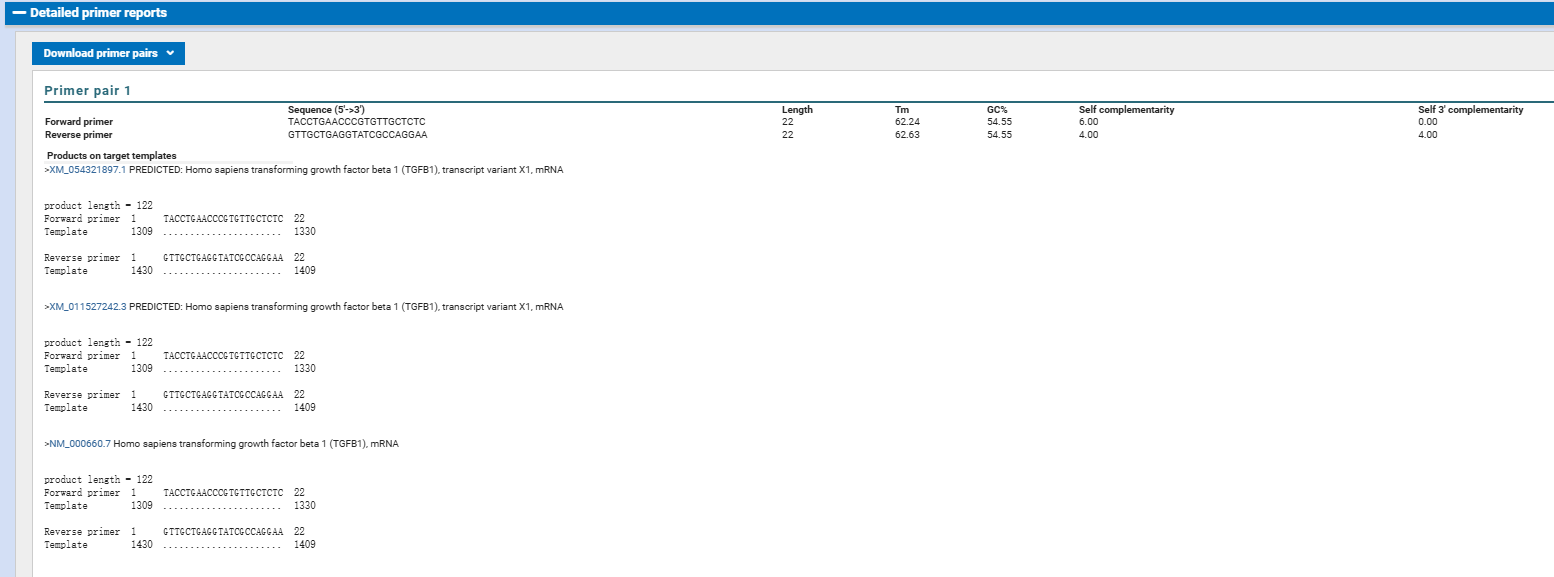


The Amplification curves:


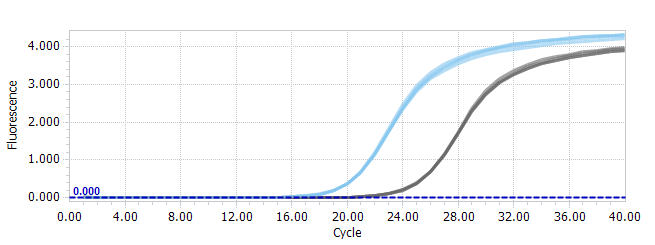


**GAPDH Human qPCR Primer Pair**

Sequence accession number:

NM_002046, NM_002046.1, NM_002046.2, NM_002046.3, NM_002046.4, NM_002046.5, BC009081, BC009081.1, BC001601, BC004109, BC013310, BC020308, BC023632, BC025925, BC026907, BC029340, BC029618, BC083511, BE893087, BG724119, BI463134, BM763361, BT006893, BU155402, NM_002046.7

The primer seqence is from Origene, CAT#：HP205798

The sequece:

Forward: 5'- GTCTCCTCTGACTTCAACAGCG -3'

Reverse: 5'- ACCACCCTGTTGCTGTAGCCAA -3'

PCR product size：131bp

Location of amplicon: chr12:6537902+6538136

Amplicon length: 235bp

Amplicon seqence:

GTCTCCTCTGACTTCAACAGCGACCACCCTGTTGCTGTAGCCAAGTCTCCTCTGACTTCAACAGCGacacccactcctccacctttgacgctggggctggcattgccctcaacgaccactttgtcaagctcatttcctggtatgtggctggggccagagactggctcttaaaaagtgcagggtctggcgccctctggtggctggctcagaaaaagggccctgacaactcttttcatcttctaggtatgacaacgaatTTGGCTACAGCAACAGGGTGGT

The melting peak of this primer:


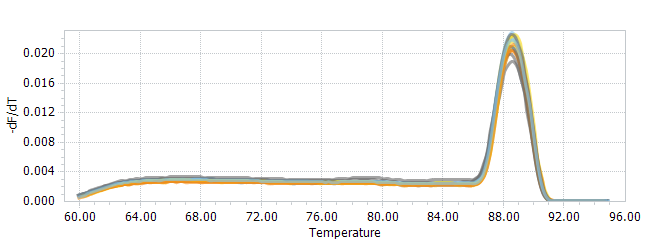


Using BLAST to silico specificity:


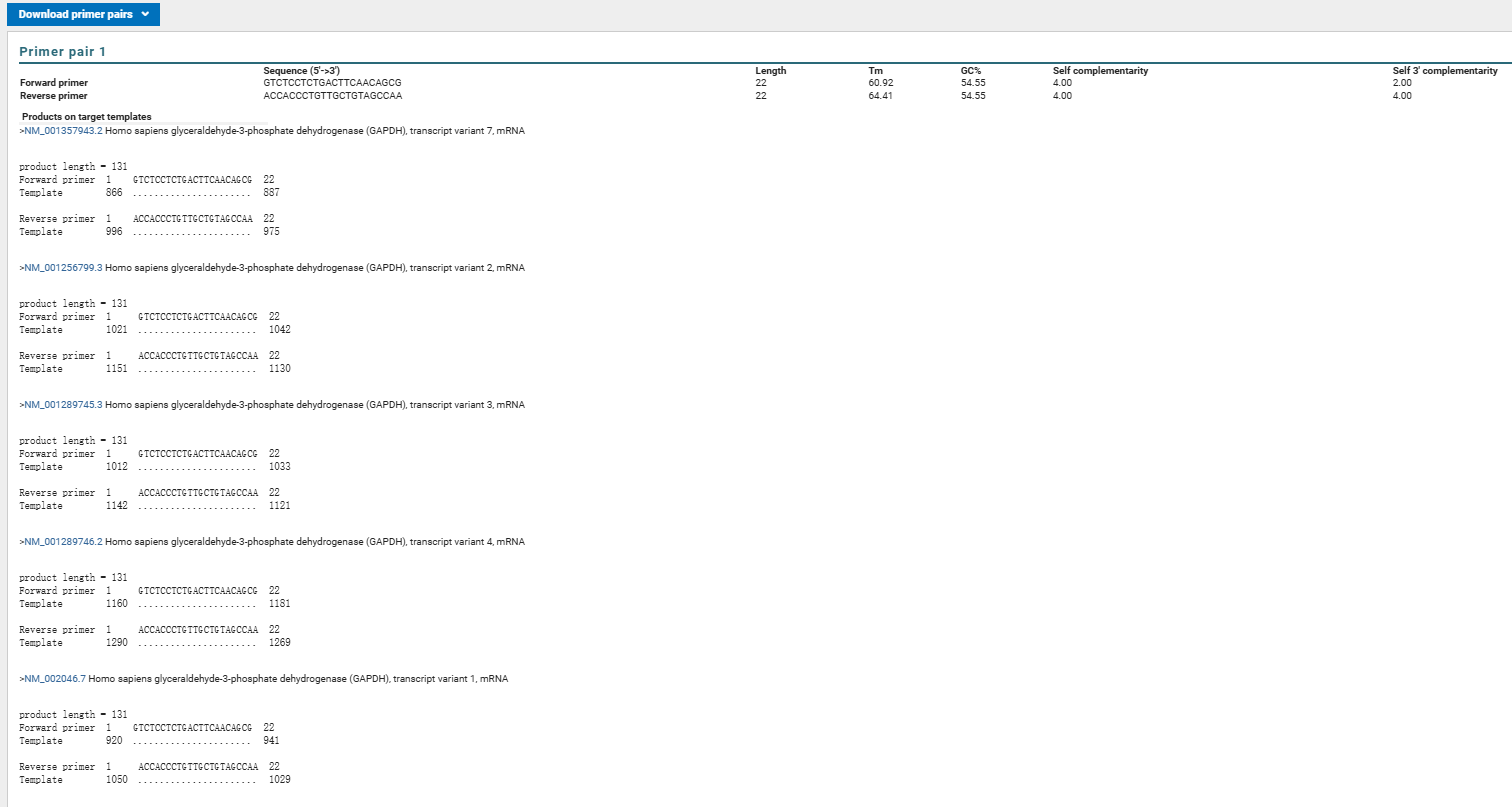


The Amplification curves:


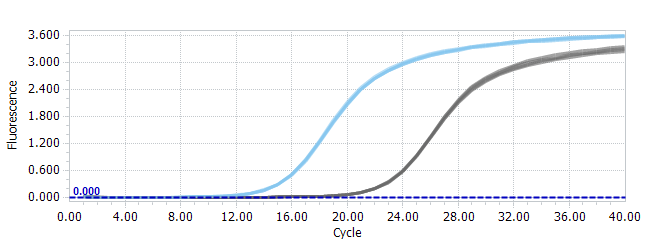

Supplement: Supplemental Information 2 [file peerj-13-19449-s002.docx]
